# Exome-Sequence Analyses of Four Multi-Incident Multiple Sclerosis Families

**DOI:** 10.3390/genes11090988

**Published:** 2020-08-25

**Authors:** Tobias Zrzavy, Fritz Leutmezer, Wolfgang Kristoferitsch, Barbara Kornek, Christine Schneider, Paulus Rommer, Thomas Berger, Alexander Zimprich

**Affiliations:** 1 Department of Neurology, Medical University of Vienna, 1090 Vienna, Austria; tobias.zrzavy@meduniwien.ac.at (T.Z.); fritz.leutmezer@meduniwien.ac.at (F.L.); barbara.kornek@meduniwien.ac.at (B.K.); paulus.rommer@meduniwien.ac.at (P.R.); thomas.berger@meduniwien.ac.at (T.B.); 2 Karl Landsteiner Institute for Neuroimmunological and Neurodegenerative Disorders, 1090 Vienna, Austria; wolfgang.kristoferitsch@meduniwien.ac.at; 3 Department of Neurology, University Medical Center Augsburg, 86156 Augsburg, Germany; Christine.Schneider@uk-augsburg.de

**Keywords:** genetics, multiple sclerosis, NGS, multi-incident family

## Abstract

Multiple sclerosis (MS) is an inflammatory demyelinating disease of the Central Nervous System (CNS). Currently, it is estimated that 30–40% of the phenotypic variability of MS can be explained by genetic factors. However, low susceptibility variants identified through Genome Wide Association Study (GWAS) were calculated to explain about 50% of the heritability. Whether familial high-risk variants also contribute to heritability is a subject of controversy. In the last few years, several familial variants have been nominated, but none of them have been unequivocally confirmed. One reason for this may be that genetic heterogeneity and reduced penetrance are hindering detection. Sequencing a large number of MS families is needed to answer this question. In this study, we performed whole exome sequencing in four multi-case families, of which at least three affected individuals per family were analyzed. We identified a total of 138 rare variants segregating with disease in each of the families. Although no single variant showed convincing evidence for disease causation, some genes seemed particularly interesting based on their biological function. The main aim of this study was to provide a complete list of all rare segregating variants to provide the possibility for other researchers to cross-check familial candidate genes in an unbiased manner.

## 1. Introduction

Multiple sclerosis (MS) is a chronic disease of the central nervous system (CNS), characterized by inflammation, demyelination, neurodegeneration, and astrogliosis. There is evidence for a genetic contribution to MS risk in epidemiological studies [1]. Cases of familial aggregation were described as early as the beginning of the 19th century [2]. Since then, family studies have convincingly shown that the risk for MS in monozygotic twins is up to 30% and up to 3% for individuals with a first-degree relative suffering from MS compared to the general population (0.1–0.3%) [3]. Within the last 15 years, great international efforts have identified over 200 genome-wide association (GWA) variants with little effect on individual risk [4,5]. Still, the molecular haplotype HLA-DRB1*15:01, described in the early 1970s, had the strongest and most consistent association with disease susceptibility [6]. Rare variants, exhibiting low frequencies in the general population (minor allele frequency < 0.5%), but exerting a stronger effect on disease susceptibility, were not detected by GWA studies due to their low frequency, which might explain the missing heritability [7]. Remarkably, a recent rare variant association study that included 32,000 MS cases suggested that up to 5% of MS heritability is explained by low-frequency variations in gene coding sequences [8]. However, the effect sizes of these variants were small (OR of ~2) and, intriguingly, they were found to be rather protective of disease. In total, rare and common variants together explain only about 50% of the estimated heritability [5,8]. Familial contribution to MS etiology has long been recognized, and many cases of families with apparent monogenic inheritance have been reported [2,9]. In the last decade, a number of putative familial high-risk genes were suggested by whole-exome sequencing (WES) studies of multi-case MS families [10,11,12]. However, none of these genes were confirmed in independent studies [10,11,12,13,14,15]. To elucidate potential putative high-risk genes, we performed WES in four MS families with three or more affected individuals and filtered for rare variants segregating with disease in the respective family. In this study, we aimed to share variants potentially implicated in MS pathogenesis and to serve as a resource for other research groups who wish to further investigate the genetic background of familial MS.

## 2. Methods and Patients

### 2.1. Participants

We clinically evaluated four multiplex families that had three or more affected family members with MS. All cases were diagnosed according to the McDonald criteria [16]. Written informed consent was obtained from all study participants. The study was approved by the local ethics committee (EK Nr: 2195/2016).

### 2.2. Sequencing

Whole-exome data were generated from 14 affected individuals from 4 families. Exomes were enriched in solution with SureSelect Human All Exon Kits 50 Mb V5 and 60 Mb V6 (Agilent, Santa Clara, CA, USA). DNA fragments were sequenced as 100 bp paired-end runs on an HiSeq 4000 system (Illumina, San Diego, CA, USA). Variants were filtered based on the minor allele frequency (MAF < 0.005), which was estimated using the Helmholtz Zentrum in-house database (>20,000 exomes) and confirmed by gnomAD (https://gnomad.broadinstitute.org). Only exonic rare variants were considered such as protein changing variants, direct splice site variants (±2 bp from exonic borders), and 5′ and 3′ UTR variants.

### 2.3. Literature Search

All single nucleotide variants (SNVs) were subjected to a literature search. We used a literature search program [17] implemented in the UCSC genome browser (http://genome.ucsc.edu/) within the subtracks “Gene,” “Gene predictions” and “Publications.”

## 3. Results

**Figure 1:**
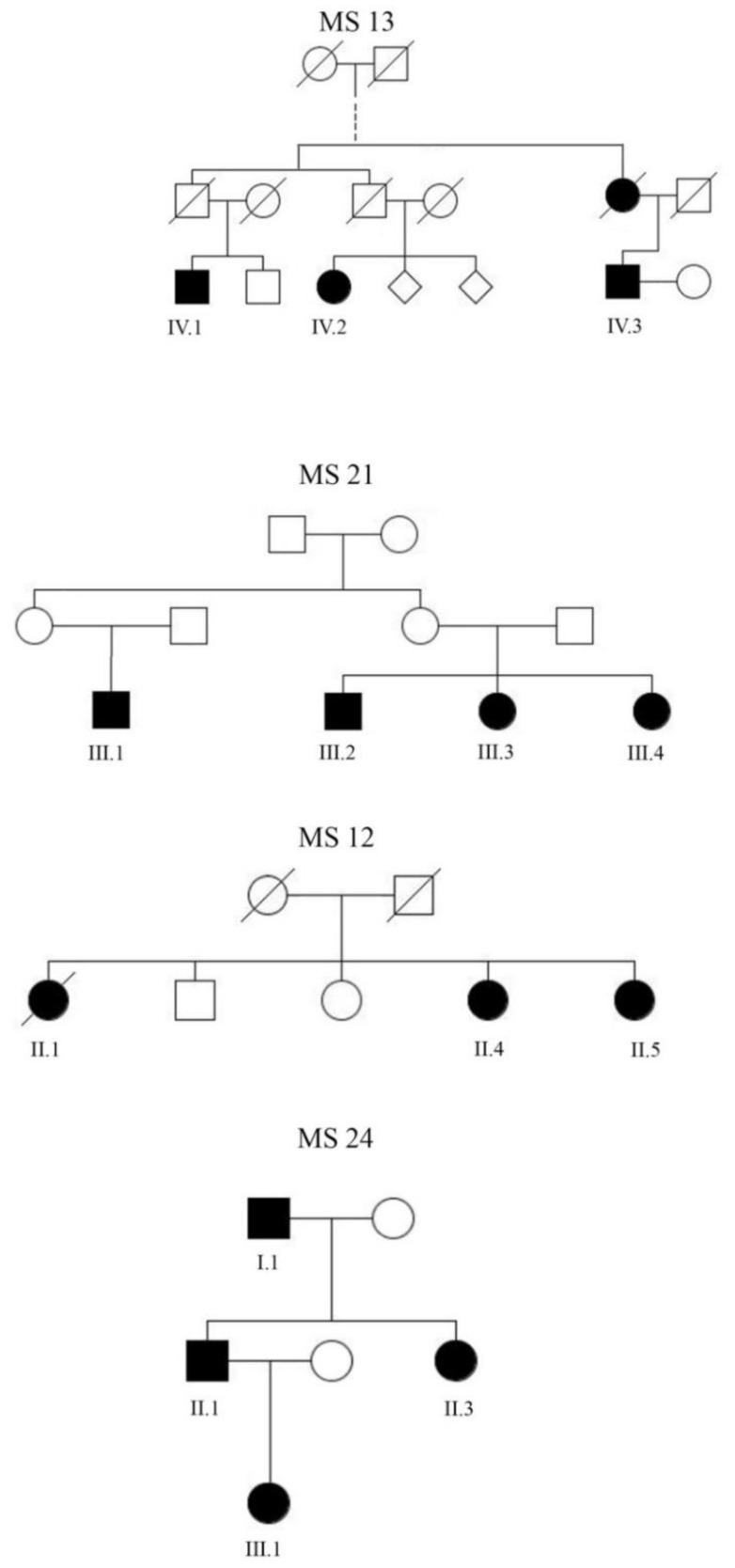
Pedigrees of the 4 families. Men are represented by squares and women by circles. A diagonal line indicates subjects known to be deceased.

We identified four MS families with three or more affected members (Figure 1). All four families were of Caucasian ethnicity and Middle European descent. Clinical details of the families are shown in Table 1. In brief, the mean age of onset was 32.5 years (range 14–50 years). All patients had an initial relapsing-remitting (RRMS) disease course. The inheritance pattern was compatible with autosomal dominant inheritance, with complete (family 24), incomplete penetrance (families 21 and 13), or recessive inheritance (family 12). Family 13 is a large family, descended from one common ancestor who lived in the 19th century. Information about disease status of members of this family before the 1950s was not available. We assumed that the disease in all of these families has a strong genetic basis and that moderate- to highly-penetrant variants might have contributed to disease development in individual family members. We performed WES in the 14 alive and affected family members (Figure 1) and filtered for rare exonic and direct splice site variants shared by all affected members in each family. We chose a relaxed frequency threshold of 0.5% (MAF < 0.005) to also capture variants conferring incomplete penetrance. We identified 138 exonic variants, including protein changing, 5′ and 3′ UTR alterations) in all four families; 1 in family 13, 90 in family 12, 6 in family 21, and 41 in family 24 (Supplementary Table S1). Surveying the literature for our 138 candidate variants revealed that none were found in previous studies to be associated with multiple sclerosis or any other disease.

## 4. Discussion

In this study, we exome-sequenced four multiplex families with MS. We could not identify a single gene variant as causative in any of the families. This was not totally unexpected, as each of the families were too small to gain a significant result, even in the hypothetical case that a singular variant was causative. In the last 10 years, the body of evidence of the underlying genetic architecture in MS families seeming to be substantially different from many other complex diseases is growing. For example, in neurodegenerative diseases, there is a significant proportion (>10%) of monogenic families in whom the disease is caused by singular highly penetrant disease genes, e.g., LRRK2 and PRKN in Parkinson’s disease or PSEN1 and APP in Alzheimer´s disease [18]. Notably, it was the high penetrance and the recurrent occurrence of the same genes in different families that enabled disease gene identification. The frequent observation of familial clustering in MS, together with a seemingly Mendelian inheritance pattern in some families, has led to the expectation that highly penetrant variants in recurrent disease genes might also be responsible for at least some cases. Disappointingly, though hundreds of multi-case families were analyzed through linkage analyses and next-generation sequencing over the last two decades, not a single unequivocally accepted locus or disease gene was identified [13,14,19,20]. Although several promising genes were nominated, none of them could be replicated in subsequent studies [13,14,21]. These observations led part of the MS genetics community to doubt the existence of high-penetrant disease genes at all [3,20]. Given the large number of family-studies that failed to detect Mendelian genes in the past, it is now commonly agreed that the existence of a highly recurrent strongly penetrant disease gene is highly unlikely. However, we think that the aforementioned studies do not necessarily contradict the existence of moderate penetrant familial genes. One reason they have not been found so far could be genetic heterogeneity and incomplete penetrance being fundamental properties underlying familial MS, which complicates gene identification. Importantly, the best proof of the bona fides of a disease gene usually comes from confirmation in different families. Therefore, we think the best method to address this problem is by providing unabridged sequencing results, which should allow other researchers to cross-check variants in an unbiased way, and thereby increase the chance that the same gene hit is found independently in other families [22]. Although we have no evidence for the pathogenicity of individual gene variants, we were able to derive some interesting observations.

In family 13, only one missense variant in the Diphthamide Biosynthesis 3 (DPH3) gene was shared by the three affected family members, DPH3-E11A. This variant is absent in all public databases. The DPH3 protein is part of the diphthamide complex, consisting of six proteins (DPH1–6). This complex mediates, in a multistep procedure, ribosylation at amino acid His-715 of the elongation factor 2 (eEF2), called diphthamide [23]. The exact physiological role of the diphthamide modification is not completely understood [23]. It is speculated that diphthamide plays a role in maintaining translational fidelity [24]. Furthermore, diphthamide protects eE2F from degradation, particularly under oxidative stress conditions. Cells missing DPH3 were found to be unable to increase translation of stress response proteins containing internal ribosomal entry sites (IRES) [25]. Intriguingly, MS pathway enrichment analyses, including 200 autosomal GWA loci, showed significant association with diphthamide biosynthesis [5]. In family 21, only two variants in the Myelin Basic Protein (MBP) and the Inositol Polyphosphate-4-Phosphatase Type II (INPP4B) genes were shared by the four affected individuals. The MPB variant (g. chr18:74700385A > T) is located in intron 5 of the canonical MBP transcript (NM_001025081.2). However, when referring to the non-canonical transcript uc010xfe.1, this variant is located within exon 4 and changes amino acid phenylalanine at position 139 to leucine (MBP-Phe139Leu). The role of MBP in the pathogenesis of MS is still unclear; it is one of the major protein constituents of the myelin sheath in the central and peripheral nervous system. One theory of MS pathogenesis postulates that the immune system is primed early in life in the periphery by pathogens, which share homologous regions with MBP; therefore, MBP could serve as an auto-antigen [26]. Furthermore, in this context, rare MBP variants show nominal association with MS in the UK biobank (*p* < 0.020) [27] (http://ukb-50kexome.leelabsg.org/). The INPP4B variant (g.chr4:143226886C > A GRCh37/hg19) is located within the intronic sequence referring to the canonical transcript, NM_003866.3. However, referring to the non-canonical transcript, uc011chp.1, the variant affects nucleotide c3 at the third base of the predicted start codon. As a consequence, this variant leads to a loss of the primary start codon ATG for methionine, which is replaced by triplet ATT for isoleucine. No other methionine, as an alternative start codon, is present within this exon. As a result, it can be assumed that translation of this transcript is severely disabled. INPP4B was recently found to be the responsible gene in a mouse model showing a combined phenotype of decreased nerve conduction velocity in the CNS and increased susceptibility to experimental autoimmune encephalitis (EAE) [28]. The same genomic region was found to overlap with human and rat MS susceptibility regions [29,30]. Also, the same locus was previously identified in an independent study as being associated with EAE severity and spinal cord injury in another mouse model [31]. Two missense variants not present in public databases were found in family 24: ThyN1 p. (Met160Thr) and NFAT5 p. (Leu1208Phe). ThyN1 has been shown to be important for B cell development [32], and transgenic mice overexpressing ThyN1 showed accelerated induction of EAE in response to myelin oligodendrocyte glycoprotein [33]. NFAT5 has been shown to be critical for the effect of sodium chloride on TH17 differentiation, thus accelerating EAE [34,35]. In an attempt to identify variants of possible higher relevance, we compared our 138 candidate genes with over 400 associated variants of a recent large meta-GWAS study [5]. We found one matching gene, TTC21A. In the GWAS analysis, an intronic TTC21A was found to be moderately associated with protection from MS disease (OR: 0.95; *p* < 1.89 × 10^−5^). In another study, TTC21A expression in lung adenocarcinoma cancer enhanced the infiltration of immune cells into tumor tissue [36]. In our study, family 12 was found to carry a heterozygous missense variant, TTC21A-Pro215Ser, which was not found in any public database.

However, the presented candidate genes must be interpreted with caution and regarded as suggestions for follow-up studies. Our patient cohort was too small to reach sufficient statistical power for any of the variants or variant combinations. Further studies, particularly including single-cell expression data from MS relevant cells such as oligodendroglia and T-cells, would be important to narrow down candidate genes.

In conclusion, in this study, we reported rare variants segregating in four multiplex families with MS in an unbiased way. In our view, reporting full data was an appropriate way to avoid misleading conclusions. By sharing our detected variants, we aim to encourage researchers to conduct similar studies, and by that joint effort, it might be possible to determine the existence or lack thereof of intermediate or high-risk variants in MS.

## Figures and Tables

**Table 1:** Patient characteristics.

Family	Patient	Age (years)	Disease Course	Age at Onset	Year of Last EDSS	EDSS	Av.Cov.	20X
MS 12	II. 1	70 *	SPMS	43	2015	9	146	98.03
	II. 4	67	RRMS	45	2020	7	134	97.71
	II. 5	63	RRMS	50	2015	6	147	96.32
								
MS 13	IV. 1	67	RRMS	31	2019	1.5	132	97.56
	IV. 2	71	SPMS	18	2015	8	218	99.18
	IV. 3	63	RRMS	42	2015	1.5	145	97.76
								
MS 21	III. 1	20	RRMS	14	2017	2	143	99.04
	III. 2	39	RRMS	31	2017	0	147	99.07
	III. 3	36	RRMS	25	2017	1	151	99.00
	III. 4	32	RRMS	24	2020	1	158	98.99
								
MS 24	I. 1	71	RRMS	48	2020	1.5	159	99.19
	II. 1	49	RRMS	35	2017	1	139	99.01
	II. 3	45	RRMS	31	2014	0	134	98.73
	III. 1	21	RRMS	18	2017	1	152	98.97

Notes: Av.Cov.: The mean average coverage of each patient’s exome dataset. 20X: Percentage of the targeted coding region which was covered with at least 20X. * deceased (2016).

## Supplementary Materials

The following are available online at https://www.mdpi.com/2073-4425/11/9/988/s1, Table S1: exonic variants of the 4 families.
