# Supplementary material for: Exome-Sequence Analyses of Four Multi-Incident Multiple Sclerosis Families
Source: Genes (Basel). 2020 Aug 25;11(9):988. doi: 10.3390/genes11090988 (PMC7563748; doi:10.3390/genes11090988)
Supplement: Supplementary file 1 [file genes-11-00988-s001.pdf]

Supplementary Table 1: Variants Family 24

genomic (GRCh37/hg19)	Gene	DNA	Protein	Exon	Function	gnomAD	dbSNP	pph2	Sift	CADD	Zygosity	Depth	% Var	gnomAD total	European (non Finnish)	Finnish	Ashkenazi	African	Latino	East Asians	South Asians	Other
10:g.15255803G>A	FAM171A1	NM_001010924.1:c.1784C>T	NP_001010924.1:p.Ser595Phe	8/8	missense	251434--4--0		benign	0.04	25.3	het	278	52	251434--4--0	113754--3--0	21612--0--0	10078--0--0	16256--1--0	34592--0--0	18394--0--0	30616--0--0	6132--0--0
10:g.75231333T>A	PPP3CB	NM_001142353.2:c.562A>T	NP_001135825.1:p.Met188Leu	5/14	missense	280032--11--0		benign possibly damaging	0.14	17.49	het	54	56	280032--11--0	128288--5--0	25076--1--0	10300--0--0	24930--0--0	34874--3--0	19798--0--0	29604--0--0	7162--2--0
10:g.84744883G>A	NRG3	NM_001165973.1:c.1022G>A	NP_001159445.1:p.Arg341Gln	11/11	missense	282338--62--0	rs117241349	benign	0.51	10.28	het	40	50	282338--62--0	128952--50--0	25080--5--0	10334--4--0	24924--2--0	35344--0--0	19940--0--0	30572--0--0	7192--1--0
10:g.105127961C>T	TAF5	NM_006951.4:c.215C>T	NP_008882.2:p.Ala72Val	1/11	missense	67512--8--0		benign	0.16	8.322	het	202	50	67512--8--0	29532--6--0	6988--0--0	2230--0--0	9652--0--0	6102--2--0	3778--0--0	7150--0--0	2080--0--0
11:g.124910494C>T	CCDC15	NM_025004.2:c.2743C>T	NP_079280.2:p.Arg915Trp	16/16	missense	271850--28--0		benign	0.01	28	het	199	45	271850--28--0	123908--19--0	24484--0--0	10134--0--0	23434--0--0	34470--1--0	19150--4--0	29276--3--0	6994--1--0
**11:g.134119062A>G**	**THYN1**	**NM_014174.2:c.479T>C**	**NP_054893.1:p.Met160Thr**	**5/7**	**missense**	**n.l.**		**possibly damaging probably damaging**	**0.02**	**24.6**	**het**	**138**	**46**	**n.l.**								
13:g.39587813T>G	PROSER1	NM_025138.4:c.1576A>C	NP_079414.3:p.Thr526Pro	11/13	missense	n.l.		benign		2.569	het	157	51	n.l.								
13:g.95363524C>T	SOX21	NM_007084.3:c.780G>A	NP_009015.1:p.Met260Ile	1/1	missense	74276--44--0		benign	0.04	22.6	het	78	56	74276--44--0	36236--20--0	6516--22--0	2674--0--0	10084--1--0	6390--1--0	3388--0--0	6862--0--0	2126--0--0
13:g.99446968G>A	DOCK9	XM_005254034.1:c.6194C>T	XP_005254091.1:p.Pro2065Leu	53/53	missense	280528--325--1		benign	0.17 0.16	12.64	het	133	46	280528--325--1	128374--221--1	25020--68--0	10352--0--0	24200--7--0	35342--18--0	35342--1--0	30576--1--0	7130--9--0
15:g.29415549T>G	FAM189A1	NM_015307.1:c.1613A>C	NP_056122.1:p.Gln538Pro	11/11	missense	180766--461--5		probably damaging	0	24.9	het	296	46	180766--461--5	73200--165--1	19916--232--3	7904--2--0	16596--4--0	24310--12--0	12266--0--0	21298--28--1	5276--18--0
15:g.42743265A>G	ZNF106	NM_022473.1:c.1136T>C	NP_071918.1:p.Leu379Pro	2/19	missense	282612--246--0	rs137908783	benign	0.16	7.254	het	148	49	282612--246--0	128958--179--0	25124--2--0	10364--0--0	24968--3--0	35432--49--0	19950--0--0	30610--0--0	7206--13--0
15:g.65345444T>C	SLC51B	NM_178859.3:c.329T>C	NP_849190.2:p.Leu110Ser	4/4	missense	282816--56--0	rs142436732	benign	0.04	21.9	het	94	47	282816--56--0	129154--50--0	25108--0--0	10370--0--0	24958--3--0	35436--3--0	19952--0--0	30616--0--0	7222--0--0
16:g.15120542T>C	PDXDC1	NM_001285445.1:c.1099T>C	NP_001272374.1:p.Phe367Leu	13/22	missense	n.l.		benign	0.72	13.48	het	312	19	n.l.								
16:g.24581399G>T	RBBP6	NM_006910.4:c.3388G>T	NP_008841.2:p.Ala1130Ser	17/18	missense	n.l.		benign	0.73	4.893	het	54	43	n.l.								
16:g.31097725A>G	PRSS53	NM_001039503.2:c.596T>C	NP_001034592.1:p.Leu199Pro	5/11	missense	n.l.		probably damaging	0.18	22.7	het	176	48	n.l.								
16:g.46772043G>A	MYLK3	NM_182493.2:c.581C>T	NP_872299.2:p.Ala194Val	3/13	missense	181988--4--0		benign	0.33	3.659	het	155	37	181988--4--0	86772--1--0	9186--0--0	5246--0--0	12848--0--0	25830--2--0	16698--0--0	20998--1--0	4410--0--0
16:g.55601350A>G	CAPNS2	NM_032330.1:c.682A>G	NP_115706.1:p.Arg228Gly	1/1	missense	248360--2--0		benign	0.17	16.1	het	193	45	248360--2--0	112260--2--0	21534--0--0	10032--0--0	15468--0--0	34466--0--0	17972--0--0	30598--0--0	6030--0--0
16:g.56535282C>T	BBS2	NM_031885.3:c.1208G>A	NP_114091.3:p.Arg403His	10/17	missense	251486--12--0	rs192007013	benign	0.53	19.03	het	74	36	251486--1--0	113764--1--0	21646--0--0	10080--0--0	16256--0--0	34592--0--0	18394--0--0	30616--0--0	6138--0--0
16:g.57789088C>T	KATNB1	NM_005886.2:c.1354C>T	NP_005877.2:p.Pro452Ser	15/20	missense	280286--382--1	rs149569503	benign	0.13	22.3	het	236	46	280286--382--1	127912--312--1	24290--43--0	10300--0--0	24772--11--0	35348--7--0	19908--0--0	30578--0--0	7178--9--0
16:g.67572594C>T	FAM65A	NM_001193523.1:c.184C>T	NP_001180452.1:p.Pro62Ser	3/22	missense	277010--69--0		benign	0.73	18.5	het	222	41	277010--69--0	125302--50--0	25024--12--0	10204--0--0	23850--0--0	35286--1--0	19686--0--0	30556--3--0	7102--3--0
16:g.69390938C>T	TERF2	NM_005652.4:c.1492G>A	NP_005643.2:p.Glu498Lys	10/10	missense	279136--377--0	rs150757154	probably damaging	0.51	34	het	106	45	279136--377--0	128074--276--0	25020--17--0	10204--0--0	24908--13--0	34358--55--0	19778--0--0	29656--2--0	7138--14--0
**16:g.69727406A>C**	**NFAT5**	**NM_006599.3:c.3624A>C**	**NP_006590.1:p.Leu1208Phe**	**12/14**	**missense**	**n.l.**		**possibly damaging benign**	**0.03**	**12.84**	**het**	**109**	**47**	**n.l.**								
16:g.72821618_72821624delCCGCCG	ZFHX3	NM_006885.3:c.10551_10556del	NP_008816.3:p.Gly3526_Gly3527del	10/10	indel	139356--78--2	rs112443847				het	229	50	139356--78--2	71090--62--1	14858--4--0	5306--4--0	13534--0--0	12170--1--0	6668--4--1	12466--1--0	3264--2--0
16:g.75301868G>C	BCAR1	NM_001170715.1:c.11C>G	NP_001164186.1:p.Pro4Arg	1/7	missense	248506--3--0		benign	0	10.36	het	52	50	248506--3--0	112516--2--0	20826--0--0	9276--0--0	21646--0--0	32624--0--0	18372--0--0	26784--1--0	6462--0--0
16:g.82101883G>A	HSD17B2	NM_002153.2:c.374G>A	NP_002144.1:p.Arg125Gln	2/5	missense	251432--3--0		benign	0.22	22.5	het	124	51	251432--3--0	113740--1--0	21646--0--0	10078--0--0	16256--0--0	34584--1--0	18382--0--0	30610--1--0	6136--0--0
17:g.3301171G>C	OR1E1	NM_003553.2:c.534C>G	NP_003544.2:p.Phe178Leu	1/1	missense	n.l.		possibly damaging	0	26.5	het	237	46	n.l.								
18:g.57020508T>C	LMAN1	NM_005570.3:c.565A>G	NP_005561.1:p.Ser189Gly	5/13	missense	n.l.		benign	1	8.51	het	113	48	n.l.								
1:g.12939694T>A	PRAMEF4	NM_001009611.4:c.1108A>T	NP_001009611.2:p.Asn370Tyr	4/4	missense	276654--6--0	rs201682396	possibly damaging	0.12	19.3	het	86	35	276654--6--0	126322--5--0	24772--1--0	10182--0--0	23878--0--0	34324--0--0	19778--0--0	30356--0--0	7042--0--0
1:g.210536249G>A	HHAT	NM_001170564.2:c.145G>A	NP_001164035.1:p.Gly49Arg	3/10	missense	282792--168--0	rs141591165	benign	0.69	15.29	het	110	46	282792--168--0	129146--158--0	25112--7--0	10368--0--0	24954--4--0	35426--7--0	19952--0--0	30614--6--0	7220-4--0
7:g.102448760C>T	FAM185A	NM_001145269.1:c.739C>T	NP_001138741.1:p.Arg247Cys	7/7	missense	188864--108--0	rs145625209	benign possibly damaging	0.05	15.86	het	72	44	188864--108--0	76600--74--0	20366--1--0	8802--23--0	16824--2--5	25518--5--0	12458--0--0	22772--1--0	5524--2--0
7:g.129107275A>C	STRIP2	NM_020704.2:c.1861A>C	NP_065755.1:p.Ile621Leu	17/21	missense	250768--1--0	rs745785530	benign probably damaging	0.09	31	het	102	57	250768--1--0	113528--1--0	21356--0--0	10070--0--0	16232--0--0	34498--0--0	18390--0--0	30582--0--0	6112--0--0
1:g.2520973C>T	FAM213B	ENST00000378427.1:c.*120C>T		7/7	3_prime_UTR_variant		rs111819834			5.094	het	304	50	31354--40--0	15404--15--0	3474--2--0	290--0--0	8694--16--0	848--1--0	1560--4--0	0--0--0	1084--2--0
10:g.44102498G>A	ZNF485	NM_001318140.1:c.-1G>A		2/5	5_prime_UTR_variant		rs201767643			16.27	het	144	49	31354--40--0	15404--15--0	3474--2--0	290--0--0	8694--16--0	848--1--0	1560--4--0	0--0--0	1084--2--0
16:g.67472349*82del	ATP6V0D1	NM_004691.5:c.*53_*82del		8/8	3_prime_UTR_variant		rs1265758032				het	52	33	31308--1--0	15388--1--0	3472--0--0	288--0--0	8666--0--0	848--0--0	1558--0--0	0--0--0	1088--0--0
18:g.12277434A>G	CIDEA	NM_001279.4:c.*165A>G		5/5	3_prime_UTR_variant		rs1461929091			0.849	het	27	48	30426--1--0	14976--1--0	3264--0--0	284--0--0	8526--0--0	786--0--0	1544--0--0	0--0--0	1046--0--0
5:g.435041G>A	AHRR	NM_001242412.1:c.*92G>A		11/11	3_prime_UTR_variant		rs557100813			0.312	het	35	43	31408--20--0	15432--14--0	3474--0--0	290--1--0	8716--1--0	848--1--0	1560--0--0	0--0--0	1088--3--0
7:g.94285513G>A	SGCE	NM_001346719.2			5utr regulation		rs1404487860			13.88	het	178	48	n.l.								
7:g.138279297G>A	SVOPL	NM_001139456.1:c.*58G>A		15/15	3_prime_UTR_variant		rs565695569			3.922	het	65	52	31336--16--0	15412--12--0	3448--0--0	290--0--0	8702--3--0	842--1--0	1558--0--0	0--0--0	1084--0--0
13:g.77900914-4del	MYCBP2	NM_015057.5:c.-15_-4del		1/83	5_prime_UTR_variant		rs779632297				het	33	45	30496--20--0	14948--0--0	3364--0--0	282--0--0	8450--20--0	826--0--0	1556--0--0	0--0--0	1070--0--0
16:g.46598149G>C	ANKRD26P1	NR_026556.1:n.496G>C		2/17	transcribed_pseudogene					3.531	het	120	43	n.l.								
17:g.74865050G>T	MGAT5B	NM_001199172.2:c.-62G>T		1/18	5_prime_UTR_variant					3.357	het	85	49	n.l.								

gnomAD: Number of alleles--heterozygote carriers--homozygote carriers, n.l. not listed (https://gnomad.broadinstitute.org/)
pph2
CADD: Combined Annotation Dependent Depletion score (https://cadd.gs.washington.edu/)
Sift:
Zygosity: het: heterozygous, hom: homozygous
Depth: read depth of the variant at the position
%Var: precentage of reads of the alternate variant
n.l.: not listed
bold: all gene variants discussed in the manuscript

Supplementary Table 1: Variants Family 21

genomic (GRCh37/hg19)	Gene	DNA	Protein	Exon	Function	gnomAD	dbSNP	pph2	Sift	CADD	Zygosity	Depth	% Var	gnomAD total	European (non Finnish)	Finnish	Ashkenazi	African	Latino	East Asians	South Asians	Other
**18:g.74700385A>T**	**MBP**	**NM_001025101.1:c.750+66T>A**			**intron_variant**	**n.l.**	**n.a.**	**benign**	**0.02**	**0.11**	**het**	**200**	**45**	n.l.								
		**uc010xfe.1: c.417T>A**	B7Z3Y6-1:p.Phe139Leu	4/4	missense																	
**4:g.143226886C>A**	**INPP4B**	**NM_003866.3:c.424-196G>T**			**intron_variant**	**1/95788**		**benign**	**0**	**7.8**	**het**	**60**	**45**	95844--1--0	40850--1--0	5256--0--0	5694--0--0	5630--0--0	12314--0--0	8198--0--0	15018--0--0	2884--0--0
		**uc011chp.1:c.3G>T**	XP_005263380.1:p.Met1?	1/13	start lost																	
1:g.158263345G>A	CD1C	NM_001765.3:c.*79G>A		6/6	3_prime_UTR_variant		rs374456855			2.509	het	92	46	282274--120--0	128852--99--0	25106--1--0	10360--0--0	24884--10--0	35376--6--0	19910--0--0	30588--1--0	7198--3--0
6:g.139094704C>T	CCDC28A	NM_015439.2:c.-108C>T		1/6	5_prime_UTR_variant		rs778077003			11.29	het	260	45	238864--1--0	108926--1--0	16622--0--0	9914--0--0	14516--0--0	34322--0--0	18060--0--0	30568--0--0	5936--0--0
7:g.142630638A>G	TRPV5	NM_019841.7:c.-82A>G		1/15	5_prime_UTR_variant		rs139060958			4.394	het	186	50	31384--60--1	15428--2--0	3476--0--0	290--0--0	8700--7--0	846--26--1	846--18--0	0--0--0	1088--7--0
chrX:149681118C>T	MAMLD1	NM_001177466.2:c.*889C>T		6/6	3_prime_UTR_variant		rs781858622			n.a.	het	260	49	121584--240--92	6695--115--49	50993--115--39	6282--0--0	10962--2--1	10962--0--0	8815--0--0	14029--0--0	3891--8--3

Supplementary Table 1: Variants Family 13

genomic (GRCh37/hg19)	Gene	DNA	Protein	Exon	Function	gnomAD	dbSNP	pph2	Sift	CADD	Zygosity	Depth	% Var	gnomAD total	European (non Finnish)	Finnish	Ashkenazi	African	Latino	East Asians	South Asians	Other
**3:g.16306352T>G**	**DPH3**	**NM_206831.2:c.32A>C**	**NP_996662.1:p.Glu11Ala**	**1/3**	**missense_variant**	**n.l.**		**probably damaging possibly damaging**	**0.02**	**29.5**	**het**	**190**	**48**	n.l.								

Supplementary Table 1: Variants Family 12

genomic (GRCh37/hg19)	Gene	DNA	Protein	Exon	Function	dbSNP	pph2	Sift	CADD	Zygosity	Depth	% Var	gnomAD total	European (non Finnish)	Finnish	Ashkenazi	African	Latino	East Asians	South Asians	Other
10:g.123923476A>C	TACC2	NM_006997.2	NP_008928.1:	1/17	missense intronic		benign	0.12	16.9	het	139	45	237670--29--0	104948--29--0	21196--0--0	9432--0--0	20714--0--0	32276--0--0	17086--0--0	25724--0--0	6294--0--0
12:g.48105483T>C	ENDOU	NM_0011724	NP_00116591	9/10	missense intronic	rs112570968	benign possibly damaging	0.81	23.7	het	75	45	282744--71--0	129110--50--0	25096--0--0	10368--0--0	24960--18--0	35424--3--0	19950--0--0	30612--0--0	7224--0--0
12:g.49993357C>T	FAM186B	NM_032130.2	NP_115506.1:	4/7	missense		benign		6.889	het	165	50	280936--53--0	128104--8--0	25018--0--0	10228--0--0	24948--1--0	35184--2--0	19900--0--0	30376--42--0	7178--0--0
12:g.65448898T>C	WIF1	NM_007191.4	NP_009122.2:	9/10	missense		probably damaging		24.4	het	92	43	250560--1--0	113490--1--0	21636--0--0	10042--0--0	16232--0--0	34382--0--0	18324--0--0	30348--0--0	6106--0--0
13:g.35734035G>C	NBEA	NM_015678.4	NP_056493.3:	22/58	missense		benign	0.09	16.81	het	178	46	223174--1--0	98830--1--0	20418--0--0	9444--0--0	13524--0--0	31664--0--0	16084--0--0	27642--0--0	5568--0--0
13:g.96515937T>A	UGGT2	NM_020121.3	NP_064506.3:	31/39	missense	rs149785094	possibly damaging	0	29.8	het	51	35	282360--120--1	128838--104--1	25096--0--0	10366--2--0	24950--1--0	35358--7--0	19948--0--0	30600--0--0	7204--6--0
14:g.21961355A>G	TOX4	NM_014828.2	NP_055643.1:	7/9	missense		benign	0.35	7.785	het	153	50	282418--27--0	128810--26--0	25112--0--0	10356--0--0	24944--0--0	35420--0--0	19954--0--0	30616--1--0	7206--0--0
14:g.22102976G>A	OR10G2	NM_0010054	NP_00100546	1/1	missense		probably damaging	0.18	22.1	het	174	44	276768--32--0	126198--29--0	24862--0--0	9884--0--0	24548--0--0	34768--1--0	19836--0--0	29634--1--0	7038--1--0
14:g.23745014G>C	HOMEZ	NM_020834.2	NP_065885.2:	2/2	missense		possibly damaging	0.27	7.663	het	272	53	277014--354--1	126736--251--1	24748--3--0	9922--20--0	24160--7--0	35136--58--0	19530--0--0	29738--0--0	7044--15--0
15:g.25940059G>A	ATP10A	NM_024490.3	NP_077816.1:	14/21	missense	rs147432497	probably damaging	0.06	26.4	het	184	39	282532--237--0	128878--161--0	25118--53--0	10358--0--0	24960--0--0	35430--0--0	19954--17--0	30616--0--0	7218--6--0
15:g.31775847G>A	OTUD7A	NM_130901.2	NP_570971.1:	14/14	missense		benign	0.09	22.5	het	42	36	n.l.								
15:g.52901454T>C	FAM214A	NM_019600.3	NP_062546.2:	6/13	missense noncoding		benign	0.29	0.001	het	51	39	n.l.								
16:g.58073910G>A	MMP15	NM_002428.3	NP_002419.1:	4/10	missense		possibly damaging	0.38	22.5	het	216	45	251140--1--0	21618--0--0	113520--0--0	10070--0--0	16224--0--0	34578--0--0	18384--1--0	30616--0--0	6130--0--0
16:g.75563835C>T	CHST5	NM_024533.4	NP_078809.2:	3/3	missense	rs148051752	probably damaging	0.19	27.5	het	445	45	280686--361--1	127620--317--1	25106--23--0	10316--2--0	24598--11--0	35378--5--0	19880--0--0	30592--1--0	7196--2--0
16:g.89620912G>T	SPG7	NM_003119.3	NP_003110.1:	16/17	missense		benign	0.68	20.8	het	120	58	n.l.								
18:g.74620491G>A	ZNF236	NM_0013060	NP_00129301	14/31	missense noncoding		benign	0.57	8.494	het	91	56	279092--3--0	128054--3--0	23974--0--0	10332--0--0	24166--0--0	35346--0--0	19522--0--0	30570--0--0	7128--0--0
19:g.308813G>A	MIER2	NM_017550.2	NP_060020.1:	11/14	missense		benign	0.05	25.2	het	137	55	280804--179--1	127870--141--0	24994--27--1	10270--0--0	24796--3--0	35344--3--0	19782--1--0	30572--0--0	7176--4--0
19:g.548979C>T	GZMM	NM_0012583	NP_00124528	4/5	missense	rs140428817	possibly damaging	0.06	22.3	het	40	65	263458--17--0	118060--4--0	23410--0--0	9704--0--0	22982--7--0	34350--1--0	19100--1--0	29120--4--0	6732--0--0
19:g.1506821C>A	ADAMTSL5	NM_213604.2	NP_998769.2:	10/12	missense		possibly damaging	0.19	23.4	het	61	56	173280-- 68--0	69918--41--0	18606--2--0	7970--10--0	16202--1--0	21712--2--0	12076--0--0	21724--10--0	5072--2--0
19:g.2115611C>T	AP3D1	NM_0012618	NP_00124875	19/32	missense		benign	0.6	19.4	het	129	57	247612--1--0	112122--1--0	21238--0--0	10000--0--0	15342--0--0	34402--0--0	17922--0--0	30568--0--0	6018--0--0
19:g.9579809C>A	ZNF560	NM_152476.2	NP_689689.2:	9/10	missense	rs61737643	benign	0.18	17.21	het	45	49	257102--503--1	118558--446--1	24374--9--0	9574--0--0	23774--17--0	29826--16--0	18682--0--0	25664--0--0	6650--15--0
19:g.11363543A>G	DOCK6	NM_020812.3	NP_065863.2:	3/48	missense		possibly damaging	0	21.3	het	104	40	248566--3--0	112518--0--0	21538--0--0	10040--0--0	15408--0--0	34458--3--0	17968--0--0	30594--0--0	6042--0--0
1:g.1222333G>A	SCNN1D	NM_0011304	NP_00112388	8/18	missense 5utr	rs145306516	benign	0.79	7.488	het	122	47	278760--105--0	126446--73--0	24948--8--0	10184--0--0	24436--10--0	35302--7--0	19772--0--0	30540--4--0	7132--3--0
1:g.2938266G>A	ACTRT2	NM_080431.4	NP_536356.3:	1/1	missense	rs141283101	benign	0	0.056	het	63	38	281666--53--0	128492--24--0	25088--1--0	10276--0--0	24742--4--0	35366--15--0	19940--0--0	30578--7--0	7184--2--0
1:g.3413612G>C	MEGF6	NM_001409.3	NP_001400.3:	28/37	missense intronic		benign	0.48	10.04	het	90	48	188962--209--0	76526--167--0	18134--1--0	8740--1--0	17100--9--0	26358--18--0	13292--0--0	23332--7--0	5480--6--0
1:g.12785721G>A	AADACL3	NM_0011031	NP_00109664	4/4	missense	rs185949240	possibly damaging	0.02	22.5	het	125	45	280702--132--0	128466--119--0	25036--5--0	10352--0--0	24198--3--0	35372--1--0	19534--0--0	30602--3--0	7142--1--0
1:g.17273371C>T	CROCC	NM_014675.3	NP_055490.3:	17/37	missense	rs41267493	benign	0.13	25.6	het	97	39	197826--556--0	81512--196--0	19602--2--0	8862--157--0	18416--6--0	26380--24--0	14102--0--0	23260--158--0	5692--13--0
1:g.87043695A>G	CLCA4	NM_012128.3	NP_036260.2:	12/14	missense		benign	0.1	8.009	het	132	44	247420--3--0	111856--0--0	21444--0--0	10018--0--0	15412--2--0	34304--0--0	17884--0--0	30522--1--0	5980--0--0
22:g.22040839A>G	PPIL2	NM_148175.2	NP_680480.1:	11/21	missense		benign	0.28	21.3	het	165	45	n.l.								
22:g.25255728A>G	SGSM1	NM_0010399	NP_00103503	9/26	missense		benign	0.22	18.53	het	219	49	n.l.								
22:g.31971368C>G	SFI1	NM_0010074	NP_00100746	10/33	missense noncoding		probably damaging possibly damaging	0.07	23.1	het	29	55	n.l.								
22:g.50648628C>T	SELO	NM_031454.1	NP_113642.1:	4/9	missense 5utr	rs543629121	possibly damaging	0.05	23.9	het	62	44	247564--17--0	111874--4--0	21442--0--0	9990--0--0	15274--0--0	34446--5--0	17934--2--0	30590--4--0	6014--2--0
2:g.28719012G>C	PLB1	NM_0011705	NP_00116405	1/57	missense		benign	0.56	5.662	het	99	38	251096--4--0	113434--4--0	21626--0--0	10064--0--0	16256--0--0	34588--0--0	18394--0--0	30612--0--0	6122--0--0
2:g.44055163C>T	ABCG5	XM_00526448	XP_00526453	5/12	missense 5utr	rs141828689	probably damaging	0.02	34	het	141	55	282590--370--0	128988--290--0	25076--45--0	10368--0--0	24956--3--0	35426--20--0	19950--0--0	30604--1--0	7222--1--0
2:g.109237865C>T	LIMS1	NM_0011934	NP_00118041	1/10	nonsense intronic regulation	rs56186549		0.89	35	het	190	44	182604--232--0	71648--189--0	20484--8--0	8708--0--0	15878--5--0	25482--19--0	12356--1--0	22536--2--0	5512--8--0
2:g.121991702T>C	TFCP2L1	NM_014553.2	NP_055368.1:	12/15	missense noncoding	rs139334996	benign	0.56	16.45	het	32	44	282858--655--1	129196--419--1	25094--139--0	10368--10--0	24970--12--0	35438--29--0	19952--0--0	30614--29--0	7226--17--0
2:g.175201171G>A	SP9	NM_0011452	NP_00113872	2/2	missense		benign	0.41	3.977	het	110	42	66276--3--0	23948--2--0	5680--0--0	4314--0--0	896--0--0	12604--0--0	3404--0--0	13406--1--0	2024--0--0
2:g.202508123T>C	TMEM237	NM_0010443	NP_00103785	1/13	missense 5utr regulation / Start Lost		possibly damaging	0.03	13.94	het	65	51	126266--5--0	45262--3--0	14482--0--0	7690--0--0	4228--0--0	22270--2--0	7728--0--0	21018--0--0	3588--0--0
3:g.38674658G>A	SCN5A	NM_0010994	NP_00109287	2/28	syn missense	rs571894716			7.846	het	63	52	278234--5--0	126958--1--0	24846--0--0	10288--0--0	23982--0--0	35198--4--0	19396--0--0	30474--0--0	7092--0--0
**3:g.39156160C>T**	**TTC21A**	**NM_145755.2**	**NP_665698.2:**	**6/29**	**missense noncoding**		**probably damaging**	**0.15 0.26**	**26.6**	**het**	**102**	**52**	n.l.								
3:g.49723695G>A	MST1	NM_020998 (u	Arg355Trp	8/10	missense				6.417	het	26	58	n.l.								
3:g.69244405T>C	FRMD4B	NM_015123.2	NP_055938.1:	15/23	missense intronic		probably damaging possibly damaging	0.12	26.7	het	193	53	279712--103--0	128154--81--0	24982--2--0	10304--1--0	24184--3--0	35108--14--0	19460--0--0	30420--1--0	7100--1--0
3:g.100645259C>T	ABI3BP	NM_015429.3	NP_056244.2:	2/35	missense		probably damaging possibly damaging	0.03	34	het	280	46	280306--110--2	128250--32--1	25020--4--0	10344--1--0	24182--1--0	35278--2--0	19506--0--0	30594--64--1	7132--6--0
4:g.89397119G>A	HERC5	NM_016323.3	NP_057407.2:	12/23	missense		benign	0.3	8.884	het	209	47	n.l.								
4:g.103968215A>C	SLC9B2	NM_178833.5	NP_849155.2:	7/12	missense		probably damaging possibly damaging	0.29	26.8	het	107	57	250760--3--0	113290--3--0	21620--0--0	10044--0--0	16240--0--0	34474--0--0	18386--0--0	30598--0--0	6108--0--0
5:g.137213294G>A	MYOT	NM_0013009	NP_00128784	5/11	missense	rs151094883	benign	0.04	0.166	het	174	53	282852--185--0	129164--20--0	25120--0--0	10368--0--0	24968--1--0	35436--1--0	19954--142--0	30616--16--0	7226--5--0
6:g.6175022A>T	F13A1	NM_000129.3	NP_000120.2:	12/15	missense		benign	0.47	0.008	het	119	49	n.l.								
6:g.109330635G>A	SESN1	NM_0011999	NP_00118686	1/10	missense intronic regulation	rs142983751	benign	0.1	10.54	het	36	47	87234--117--1	36916--54--0	8212--0--0	5390--1--0	9306--2--0	9088--1--0	2818--0--0	12930--48--1	2574--1--0
6:g.144835942A>C	UTRN	NM_007124.2	NP_009055.2:	36/74	missense	rs144388800	probably damaging	0.53	32	het	156	44	280598--433--0	128482--251--0	25084--84--0	10240--3--0	24948--12--0	34864--48--0	19846--0--0	29962--25--0	7172--10--0
7:g.128547443G>A	KCP	NM_0011359	NP_00112938	4/38	nonsense				11.08	het	54	41	n.l.								
7:g.149544983T>C	ZNF862	NM_0010992	NP_00109269	4/8	missense		benign	0.27	0.588	het	227	53	**n.l.**								
7:g.154429580A>G	DPP6	NM_0010393	NP_00103443	6/26	missense intronic		benign	0.1	16.9	het	139	45	191484--1--0	80434--1--0	18420--0--0	9084--0--0	12412--0--0	27240--0--0	14264--0--0	24534--0--0	5096--0--0
9:g.85627390C>A	RASEF	NM_152573.3	NP_689786.2:	5/17	missense	rs140992010	possibly damaging	0	32	het	71	45	258286--194--0	119040--1--0	24390--0--0	9692--0--0	23170--181--0	31120--8--0	17726--0--0	26536--0--0	6612--4--0
9:g.96209957T>C	FAM120AOS	NM_0013222	NP_00130915	3/3	missense noncoding	rs113476650	benign	0	3.251	het	107	53	282622--722--1	129060--446--1	25108--35--0	10366--128--0	24962--15--0	35374--54--0	19946--0--0	30594--20--0	7212--24--0
9:g.114994395G>C	PTBP3	NM_0011637	NP_00115726	10/15	missense intronic	rs144684166	benign	0.09	22.7	het	148	52	280866--66--0	128574--51--0	25064--6--0	10316--0--0	24918--0--0	34872--6--0	19810--0--0	30146--0--0	7166--3--0
9:g.121930417G>A	BRINP1	NM_014618.2	NP_055433.2:	8/8	missense	rs149284594	possibly damaging	0	26.1	het	89	48	267540--102--0	121782--71--0	24054--11--0	9404--0--0	23784--4--0	33804--5--0	19496--2--0	28366--7--0	6850--2--0
9:g.139360491C>T	SEC16A	NM_014866.1	NP_055681.1:	8/32	missense		benign possibly damaging	0.45	23.1	het	112	40	248714--3--0	112654--2--0	21484--0--0	10034--0--0	15446--0--0	34496--0--0	17964--1--0	30596--0--0	6040--0--0
9:g.139371628C>A	SEC16A	NM_014866.1	NP_055681.1:	3/32	missense		benign		23.4	het	149	51	n.l.								
9:g.140605429C>T	EHMT1	NM_024757.4	NP_079033.4:	2/27	missense intronic		benign	0.02	25.2	het	247	49	251128-24--0	113906--21--0	22508--0--0	9708--0--0	21296--1--0	32468--1--0	17880--0--0	26810--1--0	6552--0--0
X:g.30236743C>T	MAGEB2	NM_002364.4	NP_002355.2:	2/2	missense		probably damaging	0.13		het	112	46	202545-- 18--0 (6Hemiz	91462--10--0	18548--0--0	7514--0--0	18924--1--0	27688--1--0	14685--0--0	18461--6--0	5263--0--0
X:g.44929178T>A	KDM6A	NM_0012914	NP_00127834	17/29	missense noncoding		benign	1		het	293	44	n.l.								
X:g.83128179C>G	CYLC1	NM_021118.2	NP_066941.1:	4/5	missense intronic		benign	1		het	75	45	194607--94--1 (28Hemi	87707--31--0	18309--6--0	7243--0--0	18061--1--0	26816--36--1	14288--0--0	17132--3--0	5051--17--0
X:g.150912051T>C	CNGA2	NM_005140.1	NP_005131.1:	7/7	missense		benign	0.05		het	131	41	205174--7--0 (2Hemizy	92694--6--0	18537--0--0	7665--0--0	18973--0--0	28048--1--0	14846--0--0	19076--0--0	5335--0--0
13:20797308-20797309 C	GJB6	NM_0011102	NP_00110368	5/5	frameshift					het	169	53	251254--6--0	113644--5--0	21614--0--0	10064--0--0	16246--0--0	34566--0--0	18394--0--0	30600--0--0	6126--1--0
18:48723148-48723154	MEX3C	NM_016626.4:c.537_542del		1/2	inframe_deletion					het	88	39	149532--12--0	60826--2--0	9742--0--0	7602--0--0	12812--0--0	22842--0--0	9882--10--0	21074--0--0	4752--0--0
3:40457449-40457450	ENTPD3	NM_0012919	NP_00127888	7/11	frameshift					het	159	48	n.l.								
3:97499054-97499055	ARL6	NM_0013235	NP_00131044	3/9	frameshift					het	50	56	n.l.								
10:g.104849385C>A	NT5C2	NM_001351169.1:c.*44C>A		19/19	3_prime_UTR_variant	rs191195125			11.65	het	15	47	216556--9--0	100670--1--0	21492--0--0	5200--0--0	23108--0--0	26342--0--0	17520--8--0	16744--0--0	5480--0--0
11:g.429534C>T	ANO9	NM_001012302.3:c.915+36C>T			5utr intronic	rs202000265			0.412	het	151	41	273314--26--0	123754--1--0	23304--0--0	10120--0--0	24178--0--0	35054--1--0	19614--22--0	30292--2--0	6998--0--0
11:g.6584485G>A	DNHD1	NM_144666.2:c.9669+124G>A			intronic variant	rs184807755			0.91	het	167	49	180806--48--0	71814--37--0	18382--0--0	8644--1--0	16012--0--0	25444--7--0	12430--0--0	22730--0--0	5350--3--0
14:g.72205924C>T	SIPA1L1	NM_001284245.3		26/26	3_prime_UTR_variant				1.069	het	62	44	251814--456--0	117208--381--0	22706--117--0	7276--21--0	24434--5--0	30258--11--0	19146--5--0	24524--5--0	6262--11--0
17:g.3912331G>A	ZZEF1	NM_015113.4:c.8734-83G>A			intronic variant	rs753847981			2.624	het	12	50	31390--4--0	15426--3--0	3472--0--0	290--0--0	8712--1--0	848--0--0	1556--0--0	0--0--0	1086--0--0
19:g.376010C>T	THEG	NM_016585.5:c.-40C>T		1/8	5_prime_UTR_variant	rs200584728			0.909	het	14	56	137348-148-2	73586--126--2	9686--20--0	2168--0--0	17868--0--0	12464--0--0	9956--1--0	8402--0--0	3218--1--0
19:g.48182604C>T	GLTSCR1	NM_015711.3	NP_056526.3:	6/15	synonymous_variant	rs1052849514			6.9	het	117	46	59746--0--0	24890--0--0	11990--0--0	1136--0--0	3642--0--0	7352--0--0	5058--0--0	4002--0--0	1676--0--0
2:g.27579006T>C	GTF3C2	NM_001318909.1:c.-44T>C		1/19	5_prime_UTR_variant	rs777495786			11.4	het	216	53	166624--48--0	68744--10--0	8864--1--0	8608--0--0	15232--0--0	25322--3--0	12108--0--0	22494--33--0	5252--1--0
2:g.74687826G>A	INO80B	NM_031288.4			downstream_gene_variant	rs370974475			0.876	het	30	53	213504--34--0	101530--32--0	20664--0--0	4226--0--0	23472--0--0	24426--2--0	18018--0--0	15876--0--0	5292--0--0
2:g.119752106C>G	MARCO	NM_006770.3:c.*10C>G		17/17	3_prime_UTR_variant	rs111933917			4.315	het	47	45	240954--53--0	113452--0--0	22112--0--0	6544--0--0	24360--49--0	28604--4--0	18760--0--0	21156--0--0	5966--0--0
2:g.202153341A>T	ALS2CR12	NM_001127391.3:c.*37A>T		15/15	3_prime_UTR_variant	rs1396954699			6.154	het	146	54	247420--1--0	112144--1--0	21440--0--0	9836--0--0	16194--0--0	33488--0--0	18284--0--0	30010--0--0	6024--0--0
2:g.204131293C>G	CYP20A1	NM_001371702.1:c.-117C>G		4/11	5_prime_UTR_variant	rs148045812			13.84	het	201	47	282856--17--0	129166--9--0	25120--0--0	10370--0--0	24968--0--0	35440--0--0	19950--0--0	30616--8--0	7226--0--0
2:g.216003041G>A	ABCA12	NM_173076.3:c.-110G>A		1/53	5_prime_UTR_variant	rs754004029			9.716	het	124	49	31336--1--0	15382--1--0	3468--0--0	290--0--0	8702--0--0	848--0--0	1558--0--0	0--0--0	1088--0--0
3:g.49851284C>T	UBA7	NM_003335.3:c.-64C>T		1/24	5_prime_UTR_variant	rs189563401			7.814	het	138	51	31394--35--0	15418--21--0	3472--0--0	290--8--0	8718--4--0	848--1--0	1560--0--0	0--0--0	1088--1--0
3:g.180707419G>T	DNAJC19	NM_145261.4:c.-29G>T		1/6	5_prime_UTR_variant	rs181271620			11.45	het	158	45	227874--282--0	99802--222--0	20370--15--0	9206--1--0	20250--8--0	30260--2--0	16458--0--0	25336--29--0	6192--5--0
7:g.100779012C>T	SERPINE1	NM_000602.5	NP_000593.1:	7/9	syn 3utr regulation	rs756727666			12.84	het	118	42	251424--5--0	113730--1--0	21620--0--0	10080--0--0	16256--0--0	34592--0--0	18392--2--0	30614--2--0	6140--0--0
7:g.120969765G>A	WNT16	NM_016087.2	NP_057171.2:	2/4	syn 5utr	rs1210772767			12.74	het	110	44	250332--1--0	113360--1--0	21092--0--0	10062--0--0	16168--0--0	34578--0--0	18352--0--0	30610--0--0	6110--0--0
9:g.35072351C>T	VCP	NM_001354927.1:c.-196C>T		1/17	5_prime_UTR_variant	rs1462477819			19.22	het	52	48	82488--0--0	30656--0--0	4790--0--0	6380--0--0	972--0--0	16136--0--0	4034--0--0	17080--0--0	2440--0--0
9:g.112898487T>C	PALM2-AKAP2	NM_001136562.3:c.-31T>C		2/4	5_prime_UTR_variant	rs141992704			0.139	het	174	45	280270--41--0	126700--32--0	25102--0--0	10342--1--0	24968--1--0	35416--4--0	19954--0--0	30586--0--0	7202--3--0
22:g.19710735C>A	SEPT5	NM_001009939.3:c.*813C>A		11/11	3_prime_UTR_variant	rs562942429			9.871	het	46	41	161694--240--0	64980--126--0	8864--73--0	8388--16--0	14788--3--0	25204--15--0	11992--0--0	22384--0--0	5094--7--0
9:g.124751792A>G	TTLL11	NM_194252.2	NP_919228.2:	4/4	syn 3utr noncoding	rs115487738			6.898	het	125	52	282872--229--2	129192--98--1	25114--0--0	10370--0--0	24966--1--0	35438--14--0	19950--6--0	30616--96--1	7226--14--0
9:g.139306503C>T	PMPCA	NM_001282946.1:c.-173C>T		2/13	syn 5utr noncoding regulation	rs142694992			14.06	het	111	47	282752--290--0	129094--219--0	25122--10--0	10364--1--0	24948--6--0	35432--28--0	19952--0--0	30616--18--0	7224--8--0
X:g.106308898C>T	RBM41	NM_001324244.2:c.*86C>T		9/9	3_prime_UTR_variant	rs1020247920				het	149	43	21610--3--0	10719--2--0	2503--1--0	175--0--0	5847--0--0	593--0--0	987--0--0	0--0--0	786--0--0
